# How does self-efficacy, learner personality, and learner anxiety affect critical thinking of students

**DOI:** 10.3389/fpsyg.2023.1289594

**Published:** 2023-12-01

**Authors:** Jing Fu, Yi Ding, Kaihua Nie, Ghulam Hussain Khan Zaigham

**Affiliations:** ^1^School of Foreign Language, Hubei Engineering University, Xiaogan, Hubei, China; ^2^Department of Management Science, Comsats University Islamabad, Islamabad, Pakistan

**Keywords:** metacognitive learning strategies, critical thinking, academic self-efficacy, self-oriented learning perfectionism, learner anxiety, learner proactivity

## Abstract

The goal of critical thinking for students is to help them learn how to think critically and systematically so they can solve problems and make informed decisions. It aids students in developing their capacity for independent thought, allowing them to generate their own conclusions and base those decisions on facts and evidence. Therefore, one of the key goals of this study was to explore the factors affecting critical thinking of English as foreign language (EFL) learners. This article used social cognitive theory (SCT) to investigate how personal and cognitive factors affect EFL learners’ critical thinking. Data from 305 Chinese EFL learners were collected online, and structural equation modeling (SEM) was used to evaluate the data. The results showed that metacognitive learning strategies (MLS) were positively related to critical thinking and that self-efficacy, self-oriented learning perfectionism, and learner anxiety were significantly related to MLS. Moreover, MLS mediated the link between self-efficacy, self-oriented learning perfectionism, learner anxiety, and critical thinking. The findings further indicated that learner proactivity moderated the association between MLS and critical thinking. By applying social cognitive theory to examine the variables influencing EFL learners’ critical thinking, this study adds uniqueness. It does this by emphasizing the moderating influence of learner proactivity and the mediating function of metacognitive learning strategies. The findings of the research have significant ramifications for educators since they emphasize how vital it is to support metacognitive strategies for learning in order to improve EFL learners’ critical thinking abilities. Additionally, to create an atmosphere that is favorable for the development of critical thinking skills in EFL education, policymakers should think about implementing support systems and interventions that focus on learner anxiety, learner proactivity, and self-efficacy.

## Introduction

Learning a foreign language is supposed to provide settings that encourage critical thinking (CT). CT has been defined as an individual’s ability to think and draw appropriate conclusions independently (Tseng, 2019). Furthermore, critical thinking has been stated as an intentional choice to accept, reject, or defer judgment regarding a proposition, as well as the degree of assurance with which language learners accept or reject it (Ennis, 2015). To promote ESL learners’ critical thinking, ESL instructors should research or invent the most relevant teaching methods and strategies. One of the interactive methods that lecturers can use is collaborative learning strategies, in which students are required to actively participate in class discussions on any topic connected to life situations (Lau, 2015).

According to academic research, affective factors are crucial in deciding whether or not students successfully acquire a second language in the contexts of English as a second language (ESL) and English as a foreign language (EFL). Metacognitive learning strategies, which can enhance language acquisition and have an impact on critical thinking, are one of the affective factors that have been mentioned in the research. For ESL learners, critical thinking, and metacognitive learning strategies (MLS) are essential because they enable them to monitor and control their cognitive processes, develop self-awareness of the learning process, and successfully perform a variety of language activities. The main goal of the current study is to close the gap in the literature by looking at other variables that may have an impact on these variables when applied to ESL/EFL learners.

Self-efficacy, self-oriented learning perfectionism (SOP), and learner anxiety are some of the key affective factors that influence MLS and CT among ESL learners (Hayat et al., 2020). Although SOP has been stated as an important factor in learning context, its importance in relation with MLS and CT has not received much attention in the study of language acquisition, which is more closely related to psychological complications (Rhéaume et al., 2000). One more factor to be examined in this analysis is self-efficacy, which indicates people’s beliefs in their capacities to perform a task with desired outcomes, and it is an essential component of social cognitive theory (SCT). Given that it affects their beliefs in their capacity to complete different language assignments efficiently and to use suitable strategies to track and control their learning process, self-efficacy is a significant variable that impacts MLS and critical thinking among EFL learner (Razmi et al., 2020). In the field of foreign language education, the above three affective factors have been investigated from different aspects, such as the learner’s self-efficacy in writing, and the association between perfectionism and test anxiety for language learners (Stoeber et al., 2009). However, research examining MLS, critical thinking, and above-mentioned factors from the viewpoint of students is rare.

Moreover, social cognitive theory (SCT) states that individual factors are critical for defining outcome variables (Bandura, 1986). Individual personality has been identified as the key factor and boundary condition while exploring the relationship between different factors (Sun et al., 2016; Khan et al., 2019; Mehmood et al., 2022). Among the individual personalities, some scholars examined the positive relation between learner proactivity and critical thinking. Past studies have used learner proactivity as moderating variable (Kong et al., 2021). Learner proactivity fosters metacognitive learning strategies and critical thinking among EFL learners because it impacts their readiness and capacity to take responsibility and take ownership of their own learning process and achievements. Kong et al. (2021) investigated the impact of learner proactivity on self-efficacy. They discovered that learner proactivity impacted the relationship between self-efficacy and academic burnout in undergraduate nursing. As a result, learner proactivity was used as the boundary condition (as a moderating variable) in this investigation (Khan et al., 2023) of the association between metacognitive learning methods (MLS) and critical thinking. This study adds three contributions to the EFL/ESL literature. Firstly, we empirically investigated our conceptual framework using SCT theory to clarify what factors influence students’ critical thinking advancement in EFL. Secondly, in the context of EFL learning, this study incorporates the universal but understudied constructs of academic self-efficacy (SE), self-oriented learning perfectionism, learner anxiety (LA), and critical thinking. Overall, this study’s novelty lies in how it modifies the impact of learner proactivity and highlights the importance of metacognitive strategies while analyzing the factors influencing critical thinking in EFL learners through the lens of social cognitive theory. For educators, legislators, and researchers who are interested in encouraging critical thinking abilities in EFL students, the findings offer insightful information.

The summary of this study is organized as follows: Section 2 reviews previous research on key variables and theory. After that, the conceptual framework and hypotheses are introduced in section 3. The method is explained in section 4. Section 5 then presents the data analysis and testing results for the hypotheses. This paper concludes by discussing the implications for theory and management, along with a few limitations and future research areas.

## Literature review

### Social cognitive theory

According to SCT, which has been used in psychology, education, and communication, the components of an individual’s active learning process are strongly influenced by monitoring others in the context of social interactions, experiences, and outside societal factors (Bandura, 1986; Xiongfei et al., 2020). Albert Bandura proposed this theory as an extension of his social learning theory. With the incorporation of cognitive approaches to learning, a better balance has emerged between the behaviorist perspective and cognitive science, which is now based on behavioral psychology (Bandura, 1986).

We used SCT as our theoretical basis as both social cognitive theory and critical thinking highlight the significance of cognitive processes in shaping human behavior. Critical thinking requires the use of cognitive processes to evaluate and assess information, whereas social cognitive theory proposes that cognitive procedures are utilized in learning through observation and modeling. Both notions point out the significance of cognitive processes in influencing human behavior, as well as the necessity to nurture these processes to make informed decisions and act effectively.

The key insight of SCT is that three mutually reinforcing factors influence a person’s functioning, namely, personal attributes, behavior, and environment. For example, concerning this study and according to SCT, personal attributes (SE, SOP, LA), behavior (i.e., critical thinking), and a specific environment (i.e., the context of EFL learning at college or university) would act together and consequently influence each other. SCT was used in this study to compare and identify relationships between personal attributes (SE, SOP, LA, and Learner Proactivity), behavioral (critical thinking), and environmental (i.e., the context of EFL learning at college or university) factors. In this analysis, we have proposed MLS as a mechanism between personal attributes (SE, SOP, and LA) and behavior (CT) to elaborate the mediation mechanism between the two. The SCT provides the basic justification of this mechanism as it highlights the role of cognitive processes in determining human behavior and the significance of evolving these processes to think critically and for making optimal decisions.

### The conception of self-efficacy, learners’ anxiety, and self-oriented learning perfectionism

Due to the complexity of critical thinking and the problem of describing and quantifying the constructs, researchers have examined its connection to learners’ EFL success and affective factors (Ghorban Mohammadi et al., 2013). Earlier investigations have concentrated on the association between academic self-efficacy, learner anxiety, self-oriented learning perfectionism, and critical thinking.

Self-efficacy has been characterized as people’s views in their capacities to complete a task with desired outcomes (Khan et al., 2020; Wang, 2021), and it is an essential component of social cognitive theory (SCT). Self-efficacy beliefs significantly impact many facets of modern life, including decision-making, cognitive processes, and problem-solving techniques (Dweck and Leggett, 1988). Academic self-efficacy describes students’ beliefs and opinions about their academic abilities and their confidence in their skills to accomplish academic duties (Schunk and Ertmer, 2000). Stronger self-efficacy beliefs have been associated with positive learning behaviors, improved motivation, and, ultimately, higher academic accomplishment in studies (Kong et al., 2021; Teng and Yang, 2023). A recent study on Chinese university students explored the effects of different aspects of self-efficacy beliefs on academic writing performance by applying SCT theory (Teng and Wang, 2023). In this study, we expected that the self-efficacy of EFL learners would affect academic achievement positively and indirectly enhance critical thinking through learning a foreign language. Based on SCT, we unfold the mechanism through which the self-efficacy of EFL learners affects critical thinking indirectly through metacognitive learning strategies in this study.

Besides self-efficacy, many studies demonstrate that anxiety has a detrimental impact on educational performance (Ghorban Mohammadi et al., 2013; Abbas et al., 2019a,b). To understand the connection between anxiety and educational performance in language learning, it is critical to differentiate between the role of anxiety in language learning (learner anxiety) and its part in language performance. According to MacIntyre and Gardner (1989), Learner anxiety is a sensation of stress and worries that is distinctively associated with second language situations, for example speaking, listening, and learning (p. 284). Researchers have long considered learner anxiety a specific type of anxiety that occurs when studying a second or foreign language (MacIntyre and Gardner, 1989; Azhar et al., 2018).

In addition, perfectionism (i.e., striving for total completion of tasks) is one of the most important personality variables in educational psychology in terms of being a complex multidimensional trait (Frost et al., 1990). Perfectionism is a personality trait characterized as an individual having excessively high-performance standards and a high level of critical self-evaluation. According to Hewitt et al. (Filett et al., 1991), there are two types of perfectionism: SOP, socially imposed perfectionism, and other-oriented perfectionism. In this study, the concept of self-oriented learning perfectionism was used as it emphasizes the significance of trying for perfection in terms of one’s ideals, and it is associated with having intrinsic motivation for learning. Despite the importance of individual perfectionism in enhancing critical thinking, no previous studies have measured self-oriented learning perfectionism about critical thinking. This study aimed to address this gap. Building on SCT, in this study we unfold the mechanism through which self-oriented learning perfectionism of EFL learners affects critical thinking indirectly through metacognitive learning strategies. We expect a positive role of MLS between SOP and CT.

### The mediating role of metacognitive learning strategies between affective factors and critical thinking

Since a critical thinker should be able to consider the justifications for her belief systems and take precautions to guarantee that they are sound, critical thinking should require a certain amount of metacognition (Lau, 2015). The role of MLS in mediating the association between self-efficacy and critical thinking refers to how MLS can alter the connection between a person’s belief in their ability to succeed (self-efficacy) and their capacity to think critically. Studies have shown that MLS has a moderating effect on students’ self-efficacy, positive emotions, and their academic performance (Hayat et al., 2020). According to SCT metacognition governs people’s cognitive processes and overall learning patterns. MLS are learning strategies in which learners actively govern their own cognitive processes. In addition to the relation between metacognitive learning strategies and critical thinking, earlier examination suggests that self-efficacy performs an important role in decision-making, cognitive processes, and problem-solving techniques (Dweck and Leggett, 1988; Zhou et al., 2023). A recent study by Teng and Yue (2022) examined Chinese university students’ metacognition, critical thinking skills, and academic writing. They stated the importance of CT in academic learning.

SOP is a capacity to set irrationally high expectations for oneself and engage in excessive self-criticism (Razmi et al., 2020). MLS can assist students with learning perfectionism by allowing them to reflect on their own thinking and increase their control over their own learning. This understanding of the learning process improves one’s individual capacity for self-control while controlling one’s own learning desire.

Finally, MLS can aid anxious learners by allowing them to reflect on their own thoughts and increase their authority over their own learning (soliemanifar et al., 2022). Additionally, MLS can help students become more adept at critical thinking by helping them focus more deliberately, reflect on what they already know versus what needs to be learned, identify flaws in their thinking, and create learning habits. The study, therefore, considers metacognitive learning methods as a mediating factor between academic self-efficacy, self-oriented learning perfectionism, LA, and critical thinking, drawing on SCT. We expect the extended model of SCT through the mediation mechanism of metacognitive learning strategies would better explain the relationship between personal attributes (SE, LA, and SOP) and behavior (CT).

### The moderating role of LP between MLS and critical thinking

Besides the affective factors, another crucial personality factor that can influence college students’ critical thinking is the learner’s proactive personality (or learner proactivity in the context of this study). Bateman and Crant (1993) defined proactivity refer to a dispositional inclination to influence one’s surroundings via personal activities. Previous research has found that proactivity strongly affects students’ MLS, learning-related emotions, and educational achievement (Hayat et al., 2020). Personality characteristics can affect language learning in exciting, difficult, and possibly unanticipated ways. Despite numerous studies on personality characteristics and proficiency in second language acquisition conducted over many years, a complete picture of the relationship between personality qualities and proficiency in second language acquisition is still lacking. As a result, learner initiative may be reflected in learning preferences, which then motivate learning strategies and provide a particular learning outcome (Heinström, 2012). For example, deep learning has been linked to personality qualities including openness, conscientiousness, and emotional stability. Deep learning shows intrinsic drive and frequently yields a positive study outcome (Heinström, 2012). To help understand how student critical thinking develops, using the SCT lens, learner proactivity was examined as a moderating component in this study as it is a significant variable for forecasting EFL learning (Kong et al., 2021). Thus, corroborating the moderating role of learner proactivity in the relationship between metacognitive learning strategies and critical thinking is necessary to comprehend the EFL learning process better.

### The present study

**Figure 1:**
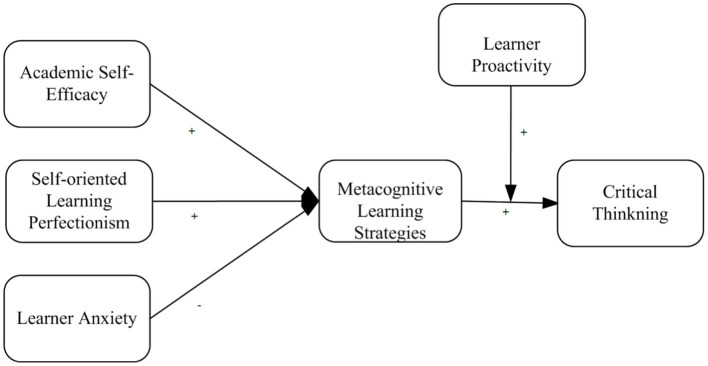
Study model.

Considering the important role of critical thinking in English language learning as mediated by metacognitive learning strategies, we examined the mediating role of metacognitive learning strategies on academic self-efficacy, self-oriented learning perfectionism, and learner anxiety on the development of critical thinking among EFL learners. Moreover, we proposed learner proactivity as a boundary condition between metacognitive learning strategies and critical thinking relationships, as shown in Figure 1.

The research questions for this study were as follows:

- Does MLS mediate the link between self-efficacy, self-oriented learning perfectionism, learner anxiety, and critical thinking in the context of EFL learning?
- Is there any association between metacognitive learning strategies and the development of critical thinking in EFL learning?
- Does learner proactivity moderate the association between metacognitive learning strategies and critical thinking in EFL learning?

Concerning these three study questions, the subsequent hypotheses were articulated:

*H1*: Academic self-efficacy has a positive relation with MLS.

*H2*: Self-oriented learning perfectionism has a positive relation with MLS.

*H3*: Learner anxiety has a negative relation with MLS.

*H4*: MLS has a positive relation with critical thinking.

*H5*: MLS mediates the relationship between (H5a) academic SE, (H5b) SOP, and (H5c) learner anxiety and critical thinking.

*H6*: Learner proactivity moderates the connection between MLS and critical thinking in such a way that this association will be stronger for students with a high level of proactivity than for those with low proactivity.

## Research methodology

### Participants and procedure

The participants in this study were Chinese adolescents who have been learning English as a second language since kindergarten. A total of 305 Chinese students were recruited through the web-based survey tool Wen Juan Xing (https://www.wjx.cn/). With the use of the online survey tool Wen Juan Xing, researchers can design, administer, gather, and evaluate data for their studies. The researchers were able to contact a greater number of subjects effectively and conveniently by using Wen Juan Xing. Because the survey tool was web-based, participants could complete it whenever it was convenient for them and there was no need for data collecting to take place in person. The sample was almost equally distributed between girls (155) and boys (150). The mean age of the participants was 18.5 years (standard deviation [SD] = 2.70), and all were studying in different colleges and universities in Central China. Ethical approval was obtained from the relevant authorities before conducting the online survey. The respondents were protected during the research process by having their details anonymized. They were aware that their involvement in the survey was entirely voluntary and that they could opt out at any time. To ensure accuracy, the questionnaires were translated into Chinese by an English language teacher and then back into English by a second English language teacher.

We utilized the Kaiser-Meyer-Olkin measure of sampling sufficiency to determine the sample size’s suitability for model validation (Hair et al., 2010; Bano et al., 2019; Khan, 2021a). This measurement produced a value for our sample size of 0.92. According to previous research a value between 0.80 and 1.0 denotes that the sample size is sufficient for testing the model. We also utilized the G*Power analysis (Faul et al., 2007), which assessed sample adequacy at a value of 0.15 and a threshold for significance of 0.05, to further establish sample size sufficiency. According to the G* Power test, a sample size of 305 participants was sufficient to examine the impact of independent factors on variables (critical t = 1.99, *p < 0*.05). These results lead us to the conclusion that the sample of 305 participants.

### Instruments

Unless otherwise specified, a five-point Likert scale, ranging from 1 (“strongly disagree”) to 5 (“strongly agree”), was used to measure the learners’ responses. This study used five-point Likert scale as these are easy to understand and use for both survey administrators and respondents. Moreover, five-point Likert scales provide a good balance between reliability and validity and are widely used in research (Khan and Ali, 2018; Pitafi et al., 2020a,b; Xiongfei et al., 2020), which makes it easier to compare results across different studies (Cao et al., 2018; Raza et al., 2020; Khan and Khan, 2021; Ali and Khan, 2023).

*Academic SE* was measured using a four-item scale taken from the study of (Artino et al., 2010). A sample item included was: “Even in the face of difficulties, I am certain I can learn the material presented in the English learning course.” The instrument showed satisfactory internal reliability (α = 0.86). *SOP* was measured using the Almost Perfect Scale-Revised (APS-R) standard subscale (Slaney et al., 2001). A sample item of this seven-item scale included: “I have a strong need to strive for excellence.” This scale showed good internal reliability (α = 0.91). We used a four-item English LA scale developed by Hong et al. (2014) to measure LA concerning English language learning. One of the sample items included: “I worry that my proficiency in English will affect my English learning.” The internal reliability (α = 0.85) of this scale was acceptable.

A scale to measure *MLS* was taken from a previous study (Pintrich and De Groot, 1990) involving 13 Likert-type items on a 5-point scale (with “1” equivalent to “not at all true of me” and “5” equivalent to “very true of me”). One example of a statement from the scale included: “When I study English, I put important ideas into my own words.” The scale showed acceptable internal reliability (α = 0.95). *Critical thinking* was measured using a 10-item scale developed by Erawan (2010). The sample items from the ten-item scale included: “have related thinking and reasonable thinking.” This scale proved to have satisfactory internal reliability (α = 0.94). *Learner proactivity* was measured using a four-item scale created by Ashford and Black (1996). One example of the sample items included: “I am always looking for better ways to do things.” The internal reliability (α = 0.87) for this scale was adequate.

### Data analysis

By analyzing the skewness (Sk) and kurtosis (Ku) values, we first verified that our data had a normal distribution. As in previous research (Khan et al., 2021; Ju and Wang, 2023), the values were below the suggested cut-offs (|Sk| < 2 and |Ku| < 7), indicating that the normalcy assumptions were met by our data. Table 1 shows the correlations among the main variables of this study. The patterns of the relationships among the variables followed the hypothesized direction. We used confirmatory factor analysis (CFA) to verify the latent constructs. Structural equation modeling (SEM) was used to address the study hypotheses. SEM is a statistical technique for examining complex associations between variables. While SEM is frequently employed in social science research (Xiongfei et al., 2019; Khan, 2021b; Li and Khan, 2022), it is also applicable in other domains, including psychology, business, and education (Mehmood et al., 2020; Pitafi et al., 2020a,b; Ali et al., 2021). Research questions with several variables and intricate interactions can benefit from the application of SEM. AMOS (Version 24.0) software was used to estimate all CFA and SEM results using full information maximum likelihood estimation. The CFA results showed good model fit (normed fit index = 0.905; comparative fit index = 0.914; root mean square error of approximation = 0.059), with these values being higher than the threshold. Individual item reliability, internal consistency reliability, convergent validity, and discriminant validity were used to confirm the reflective constructs.

**Table 1:** Descriptive statistics, square roots of average variance extracted (AVE), and correlation matrix.

Constructs	Mean	SD	1	2	3	4	5	6	7	8
Age^a^	18.50	2.70	**–**							
Gender	0.50	0.51	0.03	**–**						
Academic Self-Efficacy	3.57	1.19	−0.05	−0.06	**(0.775)**					
SOP	2.56	1.21	0.09	0.01	0.42^***^	**(0.772)**				
Learner Anxiety	2.43	1.29	0.02	0.09	0.30^**^	−0.60^***^	**(0.775)**			
MLS	3.51	1.24	−0.04	−0.08	0.53^***^	−0.46^***^	−0.43^***^	**(0.787)**		
Learner Proactivity	3.90	1.40	−0.02	0.07	0.30^***^	−0.26^**^	−0.27^**^	0.40^***^	**(0.795)**	
Critical Thinking	3.76	1.11	−0.08	−0.14^*^	0.56^***^	−0.47^***^	−0.43^***^	0.62^***^	0.32^***^	**(0.791)**

a = age in years; n = 305; SOP = self-oriented learning perfectionism; MLS = metacognitive learning strategies; correlation is significant at the 0.01 level; square roots of AVE for all constructs are shown in parentheses.

Table 2 shows that the minimum factor loading was 0.667 and that the maximum value was 0.880, both of which were greater than the standard lower limit of 0.50 (Hair et al., 2010), indicating that there were no issues with individual item reliability in the analysis. If the value of a factor loading is greater than 0.40 but less than 0.50, an item can be retained if it does not affect the composite reliability (CR) and average variance extracted (AVE). Internal consistency reliability must be evaluated to determine the CR of each variable. According to Hair et al. (2010), the CR construct should be higher than 0.60. As shown in Table 2, all CR values were greater than the threshold, confirming the reliability of all constructs. Convergent validity was assessed using AVE values, as shown in Table 2, and the AVE values exceeded the acceptable range of 0.50 (Fornell and Larcker, 1981), confirming the convergent validity benchmark.

**Table 2:** Items loadings, composite reliability, and average variance extracted of all variables.

Construct	Items	Loadings	CR	AVE	Construct	Items	Loadings	CR	AVE
Metacognitive Learning Strategies (MLS)	MLS1	0.781	0.955	0.620	Critical Thinking (CT)	CT1	0.795	0.943	0.626
	MLS2	0.765				CT2	0.810		
	MLS3	0.762				CT3	0.790		
	MLS4	0.774				CT4	0.809		
	MLS5	0.781				CT5	0.719		
	MLS6	0.760				CT6	0.735		
	MLS7	0.765				CT7	0.858		
	MLS8	0.751				CT8	0.725		
	MLS9	0.864				CT9	0.826		
	MLS10	0.793				CT10	0.832		
	MLS11	0.831			Self-Oriented Learning Perfectionism (SOP)	SOP1	0.740	0.911	0.596
	MLS12	0.764				SOP2	0.826		
	MLS13	0.835				SOP3	0.787		
Academic Self-Efficacy (SE)	SE1	0.821	0.858	0.601		SOP4	0.817		
	SE2	0.799				SOP5	0.770		
	SE3	0.745				SOP6	0.778		
	SE4	0.730				SOP7	0.677		
Learner Anxiety (LA)	LA1	0.838	0.857	0.657	Learner Proactivity (LP)	LP1	0.751	0.872	0.632
	LA2	0.880				LP2	0.809		
	LA3	0.852				LP3	0.751		
	LA4	0.861				LP4	0.863		

CR, composite reliability, AVE = average variance extracted; all factor loadings are significant at the *p* < 0.001 level.

We also found that the AVE of each variable was larger than the sum of its correlations with the other constructs (Fornell and Larcker, 1981), indicating sufficient discriminant validity. This study also investigated the variance influence factors (VIF), which were found to be below the recommended value of 10 (highest VIF = 1.98), indicating that there was no serious problem with multicollinearity (Hair et al., 2010). Reliability, convergent validity, and discriminant validity measurements were sufficient. Moreover, we applied Harman’s single-factor technique to assess CMB in the data. The results showed that the first factor had only 36.5% of the total variance, which was lower than the threshold of 50, confirming that there was no issue with CMB (Bahadur et al., 2020; Hui and Khan, 2022; Khan, 2022).

### Results of hypotheses testing

**Figure 2:**
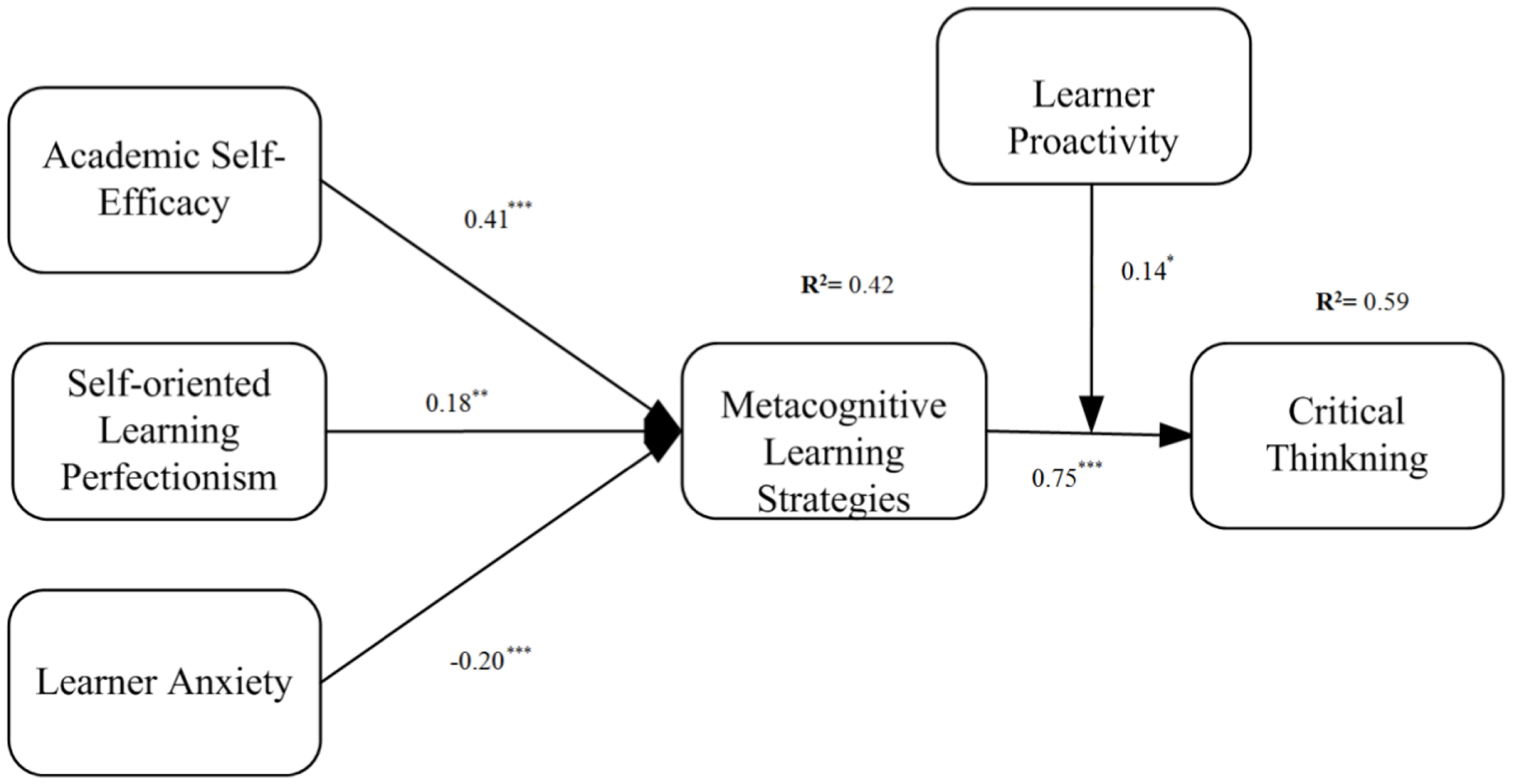
Result of path analysis; *p < 0.05, ** < 0.01, ***p < 0.001.

Hypothesis 1 stated that SE is positively related to MLS. As depicted in Figure 2 showed that academic SE was positively related to MLS (β = 0.41, *p* < 0.001), supporting hypothesis 1. Hypothesis 2 stated that SOP has a direct effect on MLS. The results showed that SOP was positively related to MLS (β = 0.18, *p* < 0.01), thus supporting hypothesis 2. Hypothesis 3 stated that LA has a direct effect on MLS. As shown in Figure 2 supported hypothesis 3 (β = −0.20, *p* < 0.01), as LA was negatively related to MLS, the variance explained by these factors into MLS was R^2^ = 0.42. Hypothesis 4 stated that MLS has a positive effect on CT, which the results showed to be the case (β = 0.75, *p* < 0.001), thus supporting hypothesis 4; furthermore, the overall variance explained in CT was R^2^ = 0.59. Moreover, age and gender were added as control variables in the studied model; however, their role was insignificant.

Following the recommendations of (Preacher et al., 2007), the bootstrapping method was applied concerning indirect effects to assess the mediation hypothesis. The findings as depicted in Table 3 showed that the following effects: SE → MLS → CT (β = 0.238, *p* < 0.001), SOP → MLS → CT (β = 0.237, *p* < 0.001), and LA → MLS → CT (β = −0.214, *p* < 0.001) were all significant. The bias-corrected 95% confidence intervals did not show any intervals straddling a zero, thus further validating the research results. Therefore, hypotheses 5a, 5b, and 5c were also supported.

**Table 3:** Bootstrap test results for the mediating relationship.

Independent variables	Effects	Bootstrap SE	Bootstrap Lower limit 95% CI	Bootstrap Upper limit 95% CI
Academic Self-Efficacy	0.238	0.041	0.165	0.326
Self-Oriented Learning Perfectionism	0.237	0.047	0.346	0.156
Learner Anxiety	−0.214	0.040	−0.300	−0.144

CI, confidence interval; bootstrap sample size = 5,000; IV = SE, SOP, and LA, MV = MLS, DV = CT.

**Figure 3:**
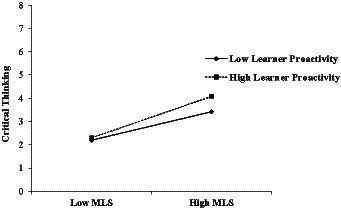
Interaction graph of metacognitive learning strategies (MLS) and learner proactivity in relation to critical thinking.

Finally, the moderating hypothesis was tested in relation to hypothesis 6, which stated that LP moderates the effect of MLS on CT. As depicted in Figure 2, the results of the moderating analysis showed an interaction effect of LP and MLS on CT (β = 0.140, *p* < 0.05), supporting hypothesis 6. We also graphically presented these effects in Figure 3. The results showed that the direct effects of MLS on CT were stronger at higher levels (β = 0.810, *p* < 0.001) of LP than at lower levels (β = 0.510, *p* < 0.001).

## Discussion

Affective factors have been put forth as important contributing factors to EFL learning. Earlier research has examined the relationship between the two (Artino et al., 2010). Others also have argued for its role in thinking (Ghorban Mohammadi et al., 2013). The present research intended to assess the impacts of self-efficacy, SOP, and learner anxiety on critical thinking and the mediating role of MLS in the link between self-efficacy, SOP, learner anxiety, and critical thinking. We also assessed the moderating effects of learner proactivity on the positive association between MLS and critical thinking.

### The potential effects of SE, SOP, and LA on critical thinking

It has been suggested that self-efficacy might influence EFL students’ critical thinking in some way. According to the literature review, many studies have been conducted on CT, self-efficacy, and learning techniques, as well as their correlations with numerous variables. However, very little information was discovered, particularly among Chinese EFL learners, regarding the issue of whether the three factors are interrelated or not and how the association between them can be considered. The findings of this research showed that Chinese EFL learners’ CT skills are significantly influenced by their sense of self-efficacy (Schunk and Ertmer, 2000).

These findings are consistent with data taken from prior investigations. For example, Fahim and Nasrollahi-Mouziraji (2013) discovered a significant positive association between leaners’ academic self-efficacy and their CT inclinations. Past studies have shown that perfectionism is a significant personality trait that influences the language performance of Chinese college students: the more perfectionism tendencies, the lower the amount of learning language skills, especially in listening and speaking. The evidence reported in this study fills the gap in the correlation between self-oriented perfectionism and critical thinking in EFL teaching, explaining at least that the interaction of metacognitive instruction and perfectionism status had a direct relation on the development of EFL learners’ critical thinking, with perfectionists being more affected by metacognitive instruction than non-perfectionists.

Based on prior findings, it may be stated that if we wish to improve the language abilities of EFL learners or reduce their anxiety, we must enhance their critical thinking. But the current finding holds that LA was negatively related to metacognitive learning strategies supports the proposition that when learning a foreign language, students with high-level anxiety may feel lost and cannot use effective learning strategies to monitor the learning process and adjust themselves, which will lead to poor performance in critical thinking and language acquisition.

### The mediation of MLS between SE, SOP, LA, and critical thinking

Another finding worth highlighting is that Chinese EFL students who demonstrate self-efficacy and self-oriented perfectionist tendencies employ more metacognitive learning approaches than students who do not, and as a result, experience less anxiety when learning a new language. At this point, the critical thinking of perfectionist learners who set high expectations for themselves to promote English learning can be vastly enhanced when they have a solid and tenacious attitude toward sticking to the norms. Following prior studies, metacognitive learning strategies are related positively to critical thinking for language learning (Ku and Ho, 2010), the present investigation expands on prior research that found MLS to be a mediator between self-oriented perfectionism, self-efficacy, and critical thinking.

### The moderating role of learner proactivity between MLS and critical thinking

Finally, our findings related to our moderation hypothesis showed that learner proactivity enhanced the positive relationship between metacognitive learning strategies and critical thinking. Although no previous study has tested the moderating role of learner proactivity in the context of metacognitive learning strategies and critical thinking, this finding is similar to findings in other fields where researchers have assessed the moderating effects of individuals’ personalities in different contexts (Sun et al., 2016; Ali et al., 2019). It is important to note that motivated people are worried about the caliber of their work, both professionally as well as personally (Lebni et al., 2020; Moin et al., 2021; Meng et al., 2023), and it is essential to evaluate the effect this has on the growth of critical thinking setting and in EFL class in general.

### Implications

By applying SCT, this study clarified how affective factors associated with cognitive factors can influence EFL learners’ critical thinking. The outcome of this research is in accordance with prior research assessment of cognitive factors in the context of EFL learners. More precisely, our study’s findings showed that self-efficacy significantly moderated the relationship between self-efficacy and EFL learners’ critical thinking, accounting for the majority of the variance in MLS. This finding is consistent with SCT and shows that self-efficacy and metacognitive learning methods may be the main origins of EFL learners’ critical thinking. Learners with high levels of self-efficacy have been reported to use effective metacognitive learning strategies (Hayat et al., 2020) and such strategies enable EFL learners to think critically about making optimal present and future decisions (Halpern, 2014).

Our findings showed that self-oriented perfectionists were more likely to use several MLS that are typically related to constructive educational outcomes and demonstrate determined motivation to attain self-imposed high standards to think critically for academic success (Mills and Blankstein, 2000). Considering the direct negative relationship between learner anxiety and MLS and the related indirect effects on EFL learners’ critical thinking, teachers need to deal appropriately with students’ learner anxiety while they are working on a learning task. Overall, incorporating creative learning with minimal teacher intervention may allow learners to engage in the learning process more effectively, experience the learning benefits of the activity more comprehensively, and reduce their anxiety about the learning task more successfully (Abbas et al., 2019a,b; Aqeel et al., 2022a,b; Moin and Khan, 2023).

Finally, our results showed that LP enhanced the positive relationship between MLS and EFL learners’ critical thinking. Personality is the key element concerning learning new things and thinking critically (Mills and Blankstein, 2000). Our results suggest that parents and universities should provide psychological interventions to foster certain types of personality development among students to encourage critical thinking. Students’ interpersonal skills are also influenced by critical thinking.

### Limitations

This study has several limitations. First, only a self-reported survey was used to collect the data. Different techniques, including classroom interviews and observations, could be used in future studies to strengthen the study’s findings. Second, this study did not comprise a large sample size. The data were obtained from only one country; therefore, further studies involving larger sample sizes and data obtained from other countries would help clarify and confirm the association between the variables of interest in this study. Third, this study only examined the mediating role of metacognitive learning strategies on affective factors and critical thinking. Other potential explanatory variables could be investigated and identified in future studies (e.g., interactive classroom activities in critical thinking development). Fourth, this study did not measure causality but only correlational relationships. Finally, teacher-parent support has been investigated in student learning and stress settings (Moin et al., 2022; Wijaya et al., 2022; Aqeel et al., 2022a,b), therefore, this study suggested that future researcher may look at the possible influences of teacher-parent support on critical thinking.

## Conclusion

To sum up, this research study represents a first step toward a more thorough comprehension of critical thinking in the context of EFL. It draws attention to the necessity of learner proactivity, the influence of personal characteristics, and the importance of metacognitive strategies for learning. By taking care of these issues, educators and policymakers may help EFL students develop into self-sufficient thinkers who are capable of navigating the challenges of the contemporary world.

## Data availability statement

The raw data supporting the conclusions of this article will be made available by the authors, without undue reservation.

## Ethics statement

The studies involving humans were approved by Management committee of School of foreign language, Hubei Engineering University. The studies were conducted in accordance with the local legislation and institutional requirements. Written informed consent for participation in this study was provided by the participants' legal guardians/next of kin.

## Author contributions

JF: Conceptualization, Data curation, Formal analysis, Investigation, Resources, Validation, Writing – original draft. YD: Investigation, Supervision, Validation, Visualization, Writing – original draft. KN: Methodology, Project administration, Validation, Writing – original draft. GZ: Methodology, Software, Writing – review & editing.
