# Supplementary material for: How does self-efficacy, learner personality, and learner anxiety affect critical thinking of students
Source: Front Psychol. 2023 Dec 1;14:1289594. doi: 10.3389/fpsyg.2023.1289594 (PMC10722148; doi:10.3389/fpsyg.2023.1289594)
Supplement: Supplementary file 1 [file Table_1.docx]

| **Appendix** | |
| --- | --- |
|  | **Critical Thinking** |
| CT1 | Able to discover causes of the problems |
| CT2 | Able to tell one’s own weaknesses |
| CT3 | View the subjects in various dimensions |
| CT4 | Not to believe in anything easily |
| CT5 | Have one’s own ideas |
| CT6 | Have related thinking and reasonable thinking |
| CT7 | Provide data of estimating situations |
| CT8 | Have the work plan |
| CT9 | Able to judge one’s own deeds |
| CT10 | Select what is beneficial to oneself |
|  | **Metacognitive Learning Strategies** |
| MLS1 | When I study English for a test, I try to put together the information from class and from the book. |
| MLS2 | When I do homework, I try to remember what the English teacher said in class so I can answer the questions correctly. |
| MLS3 | It is hard for me to decide what the main ideas are in what I read. (*R) |
| MLS4 | When I study English, I put important ideas into my own words. |
| MLS5 | I always try to understand what the English teacher is saying even if it doesn't make sense |
| MLS6 | When I study for an English test I try to remember as many facts as I can. |
| MLS7 | When studying English, I copy my notes over to help me remember material. |
| MLS8 | When I study for an English test I practice saying the important facts over and over to myself |
| MLS9 | I use what I have learned from old English homework assignments and the textbook to do new assignments. |
| MLS10 | When I am studying an English topic, I try to make everything fit together. |
| MLS11 | When I read material for English class, I say the words over and over to myself to help me remember. |
| MLS12 | I outline the chapters in my English book to help me study. |
| MLS13 | When reading English I try to connect the things I am reading about with what I already know. |
|  | **Self-oriented Learning Perfectionism** |
| SOP1 | I have high standards for my performance at school. |
| SOP2 | If you don't expect much out of yourself, you will never succeed. |
| SOP3 | I have high expectations of myself. |
| SOP4 | I set very high standards for myself. |
| SOP5 | I expect the best from myself. |
| SOP6 | I try to do my best at everything I do |
| SOP7 | I have a strong need to strive for excellence. |
|  | **Learner Anxiety** |
| LA1 | I feel anxious when I learn English. |
| LA2 | I worry about my proficiency of English will affect my English learning. |
| LA3 | Using new device to learn English makes me nervous. |
| LA4 | I worry about forgetting how to use English vocabulary correctly when I have to speak out. |
|  | **Academic Self-efficacy** |
| SE1 | Even in the face of difficulties, I’m certain I can learn the material presented in the English learning course |
| SE2 | I’m confident I can do an outstanding job on the activities in the English learning course |
| SE3 | I’m certain I can understand the most difficult material presented in the English learning course |
| SE4 | Even with distractions, I’m confident I can master the clinical reasoning skills required in the English learning course |
|  | **Learner Proactivity** |
| LP1 | Nothing is more exciting than seeing my ideas turn into reality |
| LP2 | No matter what the odds, if I believe in something, I will make it happen |
| LP3 | I am always looking for better ways to do things |
| LP4 | If I believe in an idea, no obstacle will prevent me from making it happen |
